# Understanding Parental Emotions: Children's Dental Visits and the Separation Experience

**DOI:** 10.1155/2024/5893717

**Published:** 2024-11-14

**Authors:** Ruba Odeh, Carel Brigi, Tarun Walia, Raghad Hashim

**Affiliations:** ^1^Clinical Sciences Department, College of Dentistry, Ajman University, Ajman, UAE; ^2^Basic Medical and Dental Sciences Department, College of Dentistry, Ajman University, Ajman, UAE

**Keywords:** parental separation, pediatric dentistry, United Arab Emirates

## Abstract

**Objectives:** The presence of parents during dental treatment in children is a controversial concern in dental practice. This is because of conflicting views and practices regarding the presence of parents. Therefore, the objective of this study was to evaluate parental response to their presence/absence during their child's dental treatment and to determine the factors that would influence their decision.

**Methods:** A cross-sectional survey questionnaire was administered to United Arab Emirates (UAE) parents. A total of 240 parents participated in the survey, which contained 15 questions that analyzed the participants' demographic details, dental procedures influencing parental separation, and factors influencing their desire to be present during their child's dental treatment.

**Results:** Data were analyzed using descriptive statistics and chi-square tests (*p*  < 0.05). The majority (78%) of the parents chose to stay with their children during dental treatment, with a higher prevalence of female parents. The results showed that more parents opted to be with their children during invasive procedures. Younger parents are more likely to stay with their children during dental treatment. The factors influencing parental presence/absence in the dental treatment room depended on the age and nationality of the parent and the type of dental procedure (*p*  < 0.05).

**Conclusions:** Dental practitioners must provide parents with sufficient opportunities to be present during their children's dental procedures. The factors influencing parental presence/absence should be considered before deciding whether to include or exclude parents in the dental treatment room.

## 1. Introduction

Parental presence/absence, also known as the parent-in-parent-out technique in the dental office, is a controversial aspect of pediatric dentistry [1]. Some authors suggest that parental presence would be helpful to gain emotional support, eliminate separation anxiety, increase child cooperation, and increase parental satisfaction [2, 3]. However, a systematic review by De Luca et al. [4] concluded that the presence of parents during dental treatment had no effect on the child's behavior, fear, and anxiety, with little available evidence. In contrast, various studies suggest that parents are unhelpful when present during dental treatment. These include elevating parental anxiety and distress levels, potential disturbance to the work of the dentist, and the possibility of increasing child behavioral problems [5, 6].

A survey conducted by Arathi and Ashwani [7] and Peretz and Zadik [8] showed that 78.3% and 70.2% of parents expressed willingness to be present in the dental clinic, respectively. In contrast to the studies mentioned above, surveys of dentists in Minnesota [9] and the study conducted by Roder, Lewis, and Law [10] reported that dentists preferred parents to be absent from the operatory while receiving dental care. A national survey of pediatric dentists in 1989 stated that 51% of participants agreed strongly and generally that parents be absent in the dental operatory room [11].

Since parental presence/absence has both opposing and supporting views, parental separation views during dental procedures must be studied in the United Arab Emirates (UAE). Hence, the purpose of the current study was to determine the attitudes of UAE parents toward separation from their children during dental treatment and to determine the factors that influence parental separation.

## 2. Methods

The data for the present study were collected from parents who had visited pediatric dental clinics at Ajman University (AU) for their children's dental appointments. The method used in this study was a random sample survey. A sample size calculation, with a significance level of 0.05 *α* and 0.2 *β* (giving the study a power of 80%), was carried out to determine the required number of participants. It was found that a total sample size of 150 subjects would be required to detect a significant mean difference. The inclusion criteria were parents of healthy children aged between 4 and 12 years old. The questionnaire was adopted and modified from previous studies on parental separation during dental treatments [12–16]. Questions were prepared in both Arabic and English. The questionnaire consisted of three components: sociodemographic characteristics of the parents and their child, dental procedures influencing parental separation, and factors influencing their desire to be present during their child's dental treatment (Table 1). The ethical approval for this study was obtained from the Research Ethical Committee at the College of Dentistry, AU (reference number: UGD-H-18-19-42). The data for the present study were collected between March 2019 and November 2019. The parents were informed that their participation in the study was voluntary, and informed consent was obtained from all parents. Two experts reviewed the questionnaire. Then, it was validated by four experts in the field (content validity) and 15 parents (face validity), and proper adjustments to some questions were made accordingly. Cronbach's *α* was used to assess the reliability of the questionnaire and the correlation of the questionnaire items (0.78), which measured parental preference. Data were collected by two volunteer dentists at AU with good communication skills in Arabic and other commonly spoken languages in the UAE. Descriptive statistics and Chi-square test with *p*-value were set at <0.05 for significance throughout the study. The statistical analysis software used for this study was Statistical Package for Social Sciences (SPSS) version 27 (IBM Corp., Armonk, NY, USA).

## 3. Results

### 3.1. Demographic Details

A total of 256 parents awaiting dental appointments for their children participated in this study. Of the 256 feedbacks collected, 240 responses were suitable for tabulation. Sixteen parents were excluded from the study because some of them had incomplete responses, and others were reluctant to give their consent. The mean age of the parents who participated in the study was 37 years, and a greater number of responses were obtained from female parents. The level of education for a greater number of parents was a diploma or graduate, with a leading percentage of 50. The sample characteristics of the participants are presented in Table 2. Of the respondents, 71.4% chose a female parent to be present with the child during dental treatment, and 28.6% chose a male parent. A higher percentage of the 78 parents chose to be with their child during dental treatments, with 20% being uncertain about their presence/absence, and a minority of 2% preferred to be absent in the dental treatment room with their child.

### 3.2. Dental Procedures Influencing Parental Separation

#### 3.2.1. Type of Dental Treatment

**Figure 1:**
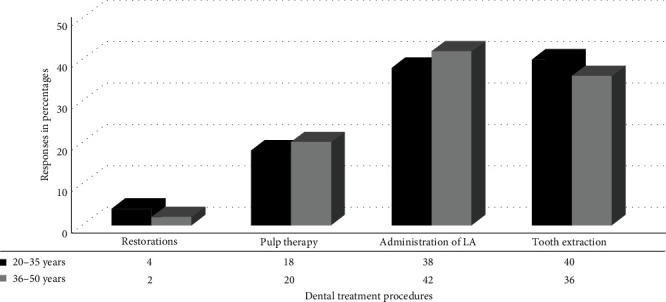
Parental presence based on the type of dental treatment. LA, local anesthesia.

Figure 1 shows the percentages of parental preferences in the dental operatory room according to dental procedures. The data showed an increased number of parents present during the administration of local anesthesia (LA) and tooth extraction procedures. Restoration procedures and pulpal therapy were the least preferred determining factors for parents to be around their children. Figure 1 also provides a table of information concerning the difference between the two age groups for each dental procedure performed. There were no statistically significant differences between the two age groups for any of the dental procedures. A chi-squared test was conducted to determine whether parental separation depended on the type of dental procedure performed. The relationship between the type of dental treatment and parental separation was significant, *χ*^2^ (3, *n* = 240) = 35.73, *p*  < 0.05.

#### 3.2.2. Dental Appointment Phase

**Figure 2:**
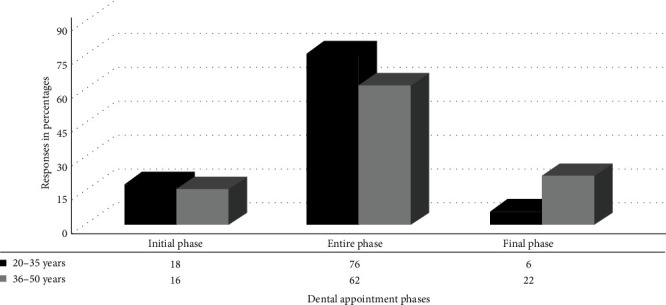
Parental presence depending on the dental appointment phase.

Figure 2 shows the preferences of the parents according to each phase during a dental appointment. Most parents opted to be present during the entire treatment phase, corresponding to 76% for the age group 20–35 years and 62% for the age group 36–50 years. Figure 2 also provides a table of information concerning the differences between the two age groups for each treatment phase. An increased percentage of parental presence was seen for the age group 20–35 years of the entire phase, and an increased percentage of parents willing to stay for the final phase was seen for the age group of 36–50 years. A chi-squared test of independence was conducted to examine the factors that would influence parents to be around their children. The association between parental decision to stay for dental treatment and the phases of treatment was significant at *χ*^2^ (3, *n* = 240) = 114.4, *p*  < 0.05.

### 3.3. Factors Influencing Their Desire to Be Present During Their Child's Dental Treatment

Table 3 shows the number of positive responses for the factors influencing parents' desire to stay with their children. Comforting their child by explaining the treatment procedure, the preference to stay when the child was uncooperative was reported by 70% and 68% of parents, respectively. Parental presence during emergencies, which would obtain information, was the least preferred. In comparing these questions about several characteristics, there were significant differences with respect to gender (*p*  < 0.05), type of dental procedure (*p*  < 0.05), and nationality (*p*  < 0.05). Differences in parental educational levels had no impact on their various responses (*p*  > 0.05). Moreover, child age groups had no impact on parental attitudes and responses (*p*  > 0.05).

## 4. Discussion

Parental presence includes benefits to both parents and dental practitioners, as stated by American Academy of Pediatric Dentistry (AAPD). Parent presence in the dental treatment room helps parents to offer physical and psychological support to young children, participate in infant examination or treatment, and observe the reality of their child's treatment. In the case of dental practitioners, it helps them to gain patient attention, enhance effective communication among the dentist child and parent, establish appropriate dentist-child roles, avert negative behavior, and gains a positive dental experience [3, 17].

The present study demonstrated that 78% of parents wanted to be present in the dental operating room regardless of the dental procedure or any specific behavior management technique used. This result was consistent with the study by Sabbagh and Sijini and [3] Marcum, Turner, and Courts [5], Abushal and Adenubi [14], and Shroff, Hughes, and Mobley [18]. This finding indicates parents' desire to visually verify their child's safety and observe the reality of the child's treatment. The current study also showed a strong preference for female parents to be in the dental treatment room. This could be due to the female parent possessing more of a protective instinct for the child's psyche than their father. Therefore, a dentist has to understand that some parents would not agree with the child being separated from them as they sense a strong feeling to protect them in the clinic [12].

Concerning the relationship between the type of dental procedure and the age characteristics of the parents in the current study, there were no significant variations between age and type of dental procedure. The most opted parental presence was during dental procedures such as tooth extraction and administration of LA. This could be due to a direct correlation between invasive dental procedures and parental presence. These findings were similar to those of studies conducted on parental presence/absence during invasive procedures [15, 19–22]. The reasons suggested for parents being present in their studies were fear stimuli. The sight of the anesthetic needle and the sound and sensation of the drill creates a stressful situation in the dental environment. Therefore, parental presence during invasive procedures benefits the child by actively supporting the dental practitioner when their child has a behavioral problem. A higher percentage of parents preferred to stay with their children during the treatment procedure. This result was similar to the studies by Sabbagh and Sijini [3] and Abushal and Adenubi [14]. There was a significant difference for older parents to be in the dental operatory room for the final phase, just to inquire about the dental procedure performed for the child. The effect of parents' age and type of dental procedure on their desire to be around their child during the dental procedure was significant in the current study. However, the above results were not in agreement with the study conducted by Kamp [23] and Peretz and Zadik [8]. The results in their study stated that age, gender, and previous dental experience had no significant correlation with parental presence in the dental operatory room. The results of this study showed that child-age groups had no impact on parental attitudes and responses. Several studies have reported that parental attitudes and responses are influenced more by individual child characteristics, parental beliefs, and situational contexts than by the child's age alone. For example, a study by Smetana, Daddis, and Chuang [24] found that while parents adjust their strategies as their children grow, the fundamental attitudes and responses remain consistent across different age groups. Furthermore, a comprehensive review by Darling and Steinberg [25] highlighted that parents often develop a set of core strategies for interaction that they apply across various age groups. These strategies are tailored to the child's individual needs and the family's overall dynamics rather than specific age-related stages [25]. Our findings support the notion that, while certain behaviors and interactions might vary with age, the underlying attitudes and responses from parents do not show significant changes attributable solely to the child's age.

The present study also indicated that 72% of parents believed that their presence during dental treatment made the child cooperative, and 68% ascertained their presence when the child was uncooperative. These results indicate that clinicians should provide parents with the opportunity to be present during their children's dental procedures. Research by Leijten et al. [26] provides evidence that the core principles of behavior management are effective across different cultural backgrounds. This meta-analysis of parent management training programs found no significant differences in outcomes based on cultural context, suggesting that techniques like “parent-in-parent-out” have the potential for global application. Many parenting challenges, such as managing children's behavior, are universal. Studies indicate that parents from different cultures face similar issues and can benefit from behavior management techniques (BMT). For instance, a study by Gardner, Montgomery, and Knerr [27] found that interventions focusing on positive reinforcement and structured discipline were effective in diverse settings, including the United Kingdom, the Netherlands, and Hong Kong. In the current study, parental preference for reinforcing BMT was limited to 59% of the parents. This study aimed to determine parental attitudes toward BMT. A lower percentage of positive responses could have been due to the dislike of parents toward their child experiencing physical or psychological distress during BMT. However, our study also demonstrated that during an emergency dental procedure, there was a decreased preference for parents to be around their children. A similar finding was observed in a study by Boie et al. [28]. The authors in their study concluded that as the level of severity increased, parents' desire to be present decreased. The statistical report also suggests that the desire to stay with a child is dependent on the nationality of the parent. The differences in variation based on nationality could be due to communication barriers. As the responses were collected from parents in the UAE, non-Arabic-speaking parents might have been concerned about their child's anxiety when the dental concerns were addressed with an Arabic-speaking dentist.

A lower percentage (42%) of parents opted that their presence in the dental operatory room would provide information about the dental procedure. This percentage confirms that parental presence in the dental operatory room provides emotional support to the child and does not distract the dentist from working intellectually. All the above findings in our study suggest open communication between parents and dentists regarding BMT. These would help to alleviate fear and anxiety and would lead to a positive attitude and build a trustful relationship between the child and the dental team [7]. Accordingly, the dental practitioner would decide the presence or absence of a parent in the operatory room that would lead to a positive or negative impact [12]. Therefore, the parental absence/presence technique has to be advocated in the dental operatory room to gain emotional support and avoid traumatic separation in younger children.

The limitation of the present study is that the survey was conducted in a university setting, as there could be variations in parental responses when collected from private clinics or hospitals. Second, the children's anxiety or behavior rating scale was not evaluated. Therefore, the study's findings should be interpreted with caution, given the limitations and that the results are applicable only to the UAE population. However, despite the limitations, the results from the current study would help dental practitioners understand the attitudes of parents regarding their separation from children during dental procedures and the factors that are dependent on it. This study provides a foundation for further research in different cultural contexts. Future studies could replicate our methodology in various regions to validate the generalizability of the “parent-in-parent-out” technique. This approach will help to establish a robust evidence base for global applicability.

## 5. Conclusions

Many of the parents who participated in the current study had a strong desire to be present with their children during dental appointments. Parental presence during dental procedures depended on the type of procedure, age of the parent, and nationality. An open conversation between the dentist and parent regarding parental presence and consideration of the factors influencing it must be assessed before including/excluding parents in pediatric dental clinics.

## Figures and Tables

**Table 1:** Parental absence/presence questionnaire.

*Child characteristics*
Age:
Sex:
*Parent's characteristics*
Age:
Sex:
a. Male
b. Female
Educational background:
a. High School
b. Graduate/Diploma
c. Postgraduate
Nationalities:
a. Emirates
b. Expats
Participating emirate:
a. Ajman
b. Dubai
c. Sharjah
d. Other
Do you prefer to stay with your child during dental treatments?
a. Yes
b. No
c. Not sure
Whom do you prefer to stay with your child?
a. Mother
b. Father
c. Caretakers
*Dental procedures influencing parental separation*
Which of the following dental procedures would make you absolutely stay with your child?
* *a. Dental restoration
b. Pulp therapy
* *c. Administration of local anesthesia
* *d. Tooth extraction
When would you like to enter with your child?
* *a. Initial phase
* *b. Entire phase
* *c. Final phase
* (Initial phase: Enter with the child to enquire about the procedure and then leave; Entire phase: Stay during the entire visit; Final phase: Enter at the end of the treatment to enquire about the dental procedure)*
*Factors influencing their desire to be present during their child's dental treatment*
Parental presence makes your child cooperative
* *a. Yes
* *b. No
* *c. Not sure
Would you prefer to stay when your child is un-cooperative
* *a. Yes
b. No
c. Not sure
Prefer the dentist to reinforce behavior management technique when your child is un-cooperative
* *a. Yes
b. No
c. Not sure
Do you prefer explaining the treatment procedure to your child?
* *a. Yes
b. No
c. Not sure
As a parent, my presence would obtain information about the procedure and technique used
a. Yes
b. No
c. Not sure
During an emergency, you prefer to stay with your child
a. Yes
b. No
c. Not sure

**Table 2:** Sample characteristics of participants.

Characteristics	Study sample			
	Frequency	Percent	Valid percent	Cumulative percent
Child's age				
4–7 years	178	74.2	74.2	74.2
8–12 years	62	25.8	25.8	100.0
Child's gender				
Male	136	56.6	56.6	56.6
Female	104	43.4	43.4	100.0
Parent's gender				
Male	72	30.0	30.0	30.0
Female	168	70.0	70.0	100.0
Parent's age				
20–35	90	37.5	37.5	37.5
36–50	150	62.5	62.5	100.0
Education level				
High school	20	8.3	8.3	8.3
Graduate/Diploma	122	50.8	50.8	59.2
Postgraduate	98	40.8	40.8	100.0
Nationality				
Emirates	105	43.8	43.8	43.8
Expats	135	56.3	56.3	100.0
Emirate				
Ajman	165	68.8	68.8	68.8
Dubai	20	8.3	8.3	77.1
Sharjah	40	16.7	16.7	93.8
Other	15	6.3	6.3	100.0

**Table 3:** Frequency and percentage of positive responses given by parents as factors influencing their presence.

Questions	Frequency	Percentage	Mean (SD)	*p* value
a. Parental presence makes your child cooperative	151	72	121 ± 6	NS
b. Would you prefer to stay when your child is un-cooperative	163	68	110 ± 6	*⁣* ^*∗*^<0.05^a,b^
c. Prefer the dentist to reinforce behavior management technique when your child is un-cooperative	96	59	56 ± 4	NS
d. Do you prefer explaining the treatment procedure to your child	168	70	118 ± 5	NS
e. As a parent, my presence would obtain information about the procedure and technique used	101	42	42 ± 4	*⁣* ^*∗*^<0.05^c^
f. During an emergency, you prefer to stay with your child	122	51	62 ± 5	*⁣* ^*∗*^<0.05^c^

*Note:* There were number of significant differences viz. ^a^gender, ^b^type of procedure, and ^c^nationality.

Abbreviations: NS, non significant; SD, standard deviation.

*⁣* ^*∗*^Pearson *Chi*-square test was significant for questions b, e, and f.

## Data Availability

Data will be available upon request through the main author.
